# Acculturation and self-rated health among Arctic indigenous peoples: a population-based cross-sectional study

**DOI:** 10.1186/1471-2458-12-948

**Published:** 2012-11-05

**Authors:** Bent-Martin Eliassen, Tonje Braaten, Marita Melhus, Ketil Lenert Hansen, Ann Ragnhild Broderstad

**Affiliations:** 1 Department of Community Medicine, Centre for Sami Health Research, University of Tromsø, Tromsø, N-9037, Norway; 2 Department of Community Medicine, University of Tromsø, Tromsø, N-9037, Norway; 3 Department of Medicine, University Hospital of Northern Norway, Harstad, N-9480, Norway

**Keywords:** Self-rated health, Acculturation, Kalaallit, Iñupiat, Sami, Inuit, Indigenous peoples, Living conditions, SLiCA

## Abstract

**Background:**

Acculturation is for indigenous peoples related to the process of colonisation over centuries as well as the on-going social transition experienced in the Arctic today. Changing living conditions and lifestyle affect health in numerous ways in Arctic indigenous populations. Self-rated health (SRH) is a relevant variable in primary health care and in general public health assessments and monitoring. Exploring the relationship between acculturation and SRH in indigenous populations having experienced great societal and cultural change is thus of great importance.

**Methods:**

The principal method in the Survey of Living Conditions in the Arctic (SLiCA) was standardised face-to-face interviews using a questionnaire. Very high overall participation rates of 83% were obtained in Greenland and Alaska, whilst a more conventional rate of 57% was achieved in Norway. Acculturation was conceptualised as certain traditional subsistence activities being of lesser importance for people’s ethnic identity, and poorer spoken indigenous language ability (SILA). Acculturation was included in six separate gender- and country-specific ordinal logistic regressions to assess qualitative effects on SRH.

**Results:**

Multivariable analyses showed that acculturation significantly predicted poorer SRH in Greenland. An increased subsistence score gave an OR of 2.32 (P<0.001) for reporting poorer SRH among Greenlandic men, while an increased score for Greenlandic women generated an OR of 1.71 (P=0.01). Poorer SILA generated an OR of 1.59 in men (p=0.03). In Alaska, no evidence of acculturation effects was detected among Iñupiaq men. Among Iñupiaq women, an increased subsistence score represented an increased odds of 73% (p=0.026) for reporting poorer SRH. No significant effects of acculturation on SRH were detected in Norway.

**Conclusions:**

This study shows that aggregate acculturation is a strong risk factor for poorer SRH among the Kalaallit of Greenland and female Iñupiat of Alaska, but our cross-sectional study design does not allow any conclusion with regard to causality. Limitations with regard to wording, categorisations, assumed cultural differences in the conceptualisation of SRH, and confounding effects of health care use, SES and discrimination, make it difficult to appropriately assess how strong this effect is though.

## Background

Acculturation is for indigenous peoples [1] related to the process of colonisation over centuries [2]. Being one of the most cited definitions [3], Redfield, Linton and Herskovits [4] define acculturation as “those phenomena which result when groups of individuals having different cultures come into continuous first-hand contact, with subsequent changes in the original culture patterns of either or both groups” (p. 149). In health research the concept of acculturation has usually been applied to assess the health effects resulting from contact between people belonging to different ethnic groups; but the concept has also shown to be useful when exploring health implications among people subjected to rapid modernization and subsequent social and cultural change [5].

Within Alaska there are some 47.000 Inuit [6]. Approximately 30% of these are Iñupiat [6-8] inhabiting the northern and western coasts as far south as Norton Sound [6]. Greenland is home to about 57.000 people, of which approximately 90% are Kalaallit (Greenlanders). The majority of Greenland’s population is situated on the south-central west coast. Only 3500 live on the east coast and less than 1000 are located in the far north. Kalaallisut (the Greenlandic language) is closely related to the Iñupiaq language spoken by Iñupiat in Alaska [9]. The traditional Sami settlement area (Sápmi) in Norway stretches from Finnmark in the north to Engerdal in Hedmark County in the south. No reliable or updated demographic record on the Sami exists. Though suffering from grave deficiencies, estimates of the total number of Sami in Norway usually vary between 40.000 and 50.000 [10].

The Iñupiat, Kalaallit and the Sami share a common, though independently unique, history of colonialism and have throughout history been victims of state and church driven forced assimilation [11-14]. Forced assimilation has resulted in loss or extensive change of traditional practices, native languages, and norms and beliefs [2]. As part of this process, concentration of the populations in large settlements provided most circumpolar indigenous peoples with schooling, health care, housing, water, sanitation, and imported foods and consumer products [14-16]. The post-World War II years in the Arctic were characterised by an intensification of social and cultural change [14]. In Greenland, Alaska, and Norway an increasing urbanisation has taken place [14] and mining, industrial fishing and the discovery of oil transformed – to a varying degree – the economies [17]. In 2005 only 17% of the Greenlandic population lived in villages [9]. In the post-war period in Norway and Alaska, outmigration from rural to urban areas has also been considerable [18,19]. Today the transition from hunting and small scale fishing to a mixed cash/harvest economy is seen all across the Arctic [16]. Subsistence and traditional foods is still a significant contributor to cultural identity and social cohesion among the Inuit and Sami [14,20,21], but unemployment is a problem in many Inuit communities which affects subsistence as this requires costly equipment such as guns, ammunition, snowmobile, and petrol [14,22-24]. The role of subsistence is also affected by access to the resources traditionally harvested being reduced by climate change, pollution, and an increasing number of regulations and import bans [16,20,22,23,25].

Changes in living conditions and lifestyle following this development affect health in numerous ways [16]. The life expectancy of the Inuit and the Sami has dramatically improved since 1950 [26-28], but the general health status of the Inuit is still inferior when compared with their respective state’s majority populations; this disparity has often been attributed to the Inuit’s relatively poorer socioeconomic status (SES) [16]. Few general health discrepancies between Sami and ethnic Norwegians are detected today. This is largely explained by little inter-ethnic variation in SES parameters [28].

The changes in occupational patterns are associated with increased acculturative stress, decreased physical activity and change in diet [16]. A number of studies on Inuit populations report a transition from the traditional and protein rich diet, to a diet of unhealthy store-bought foods thus making the Inuit susceptible to a variety of life-style diseases such as diabetes and cardiovascular disease [29]. High prevalence rates of obesity [30], and changes in diet [31] are also found in the Sami population in Norway. The effects of acculturation on chronic life-style diseases are evident in other populations and immigrant groups too [32].

Acculturative stress may be perceived as a response to life events associated with intercultural contact. To deal with various stressors, individuals will adopt different coping strategies eventually leading to some form of adaptation, of which integration may be the most health beneficial and marginalisation the least advantageous in terms of mental health [33]. The mental health effects of the various adaptations are very much debated [34]. A relationship between marginalisation and depression/anxiety was found in a study among rural Sami adolescent males [35]. Similarly, in Greenland it was found that better mental health status was associated with growing up in a town and being fully bilingual, as opposed to growing up in a small village and only speaking Greenlandic [36]. Spein et al. [37] found that more assimilated Sami adolescents reported more smoking and drinking compared with less assimilated Sami peers. Wolsko et al. found that among Alaska Yup’ik, higher levels of acculturation was associated with greater psychosocial stress, less happiness, and greater use of drugs and alcohol [38,39]. Wexler reports a relationship between loss of traditional knowledge, alcohol abuse, and low education attainment among Iñupiat in Northwest Alaska [24].

The direction of the associations in the many studies on health and acculturation is of course related to the fact that acculturation is context sensitive and that it has been operationalized differently in relation to a variety of health outcomes. Although numerous studies have explored how acculturation is related to various health outcomes, it still remains unclear how acculturation may be related to self-rated health (SRH) [40]. As summarised by Hansen et al. [41], even after a variety of physical, sociodemographic and psychosocial health status indices are controlled for [42], SRH significantly predicts mortality, and morbidity and subsequent use of health services [43]. In sum, SRH conceptually functions as a composite measure of mental and physical health [40], and becomes thus a relevant variable in primary health care and in general public health assessments and monitoring [44]. Exploring the relationship between acculturation and SRH in indigenous populations having experienced great societal and cultural change is thus of great importance.

Subsistence and traditional foods is a significant marker of Inuit and Sami culture and identity [14,20,21]. Our study thus conceptualises acculturation as certain traditional subsistence activities being of lesser importance to people’s ethnic identity, and poorer spoken indigenous language ability (SILA). Using data from the *Survey of Living Conditions in the Arctic: Inuit, Sami, and the Indigenous Peoples of Chukotka (SLiCA)*, we explored how these activities and SILA were associated with SRH by gender among the Iñupiat of Alaska, Kalaallit of Greenland, and Sami of Norway.

## Methods

SLiCA is an international research project on health and other aspects of the living conditions of indigenous peoples in Alaska, Canada, Greenland, Norway, Sweden, and Russia.

The principal method in SLiCA was standardised face-to-face interviews using a questionnaire, which may be accessed on the project website [45]. The SLiCA target population was indigenous individuals aged 16 years and older (≥15 in Greenland) residing in traditional settlement regions. Data collection in Alaska and Greenland took place from January 2002 to February 2003 and from December 2003 to August 2006, respectively. In Norway the majority of the material was collected between June 2006 and June 2008 and a smaller amount (n=67) in 2003. Very high overall participation rates of 83% were obtained in Greenland and Alaska, whilst a more conventional rate of 57% was achieved in Norway. More detailed descriptions of the material, methods, and methodological issues in SLiCA are published elsewhere [46].

Self-rated health was measured by the question: How would you describe your health in general: *Excellent*, *Very good*, *Good*, *Fair*, or *Poor*? The labelling of categories varied somewhat in Greenland, i.e. *Very good*, *Good*, *Fair*, *Poor*, *Very poor*. In Norway and Alaska the variable was in the analyses coded: 0) *Excellent*, 1) *Very good*, 2) *Good/Fair/Poor*. In Greenland SRH was coded: 0) *Very good*, 1) *Good*, 2) *Fair/Poor/Very Poor*.

Established as essential Inuit and Sami culture values and identity markers, 12 standardised ordered categorical variables (G1: a, b, c, d, f, g, h, i, k, l, n, o) [45] measuring the importance (0=*Very important* through 3=*Not at all important*) of certain traditional subsistence activities, was chosen to measure acculturation. The items were selected in advance as they were considered relevant in all three countries. A score ranging from 0 through 36 was produced by adding the 12 variables, from which respective score averages were generated. Thus, a one unit increase in score average gave an approximation of the average effect of a one unit increase in all 12 variables. An increased score average indicated stronger acculturation, i.e. the activities were of lesser importance to a person’s Sami or Inuit cultural identity. Observations providing information from less than 9 variables in the score were coded as missing.

The linear acculturation scales have been criticised [47-49] and are replaced by models [50] attempting to explore the multidirectional nature of acculturation by incorporating possible adaptations such as integration and assimilation (see discussion). SLiCA was not exclusively and specifically designed for conducting multidimensional acculturation studies; we thus had to settle for a conventional scale-based analysis. We have nevertheless addressed much of the criticism by scrutinising the 12 subsistence variables in three country/region-specific exploratory factor analyses [51], all of which convincingly pointed to a one-factor solution that systematically presented strong factor loadings (≥0.57), overall low unique item variance (<0.6), and large sized overall Kaiser-Meyer-Olkin test results (≥0.87) (data not shown). We thus considered scale analysis to be an appropriate substitute.

Additionally, spoken indigenous language ability (SILA) was included in the regression model (see below) as language represents an integral part of a person’s cultural identity [11]. In the analyses, SILA was dichotomised due to small sample sizes; and as distributions differed, the variable was dichotomised differently. The question was: How would you rate your ability to speak Inuit/Sami? In Norway and Greenland the recoding was: 0) *Very well*, 1) *Relatively well/With effort/A few words/Not at all*. In Alaska the variable was dichotomised into: 0) *Very well/Relatively well* 1) *With effort/A few words/Not at all*.

Housing condition was not included in the regression models in Norway. Overall the standard of living is high in Norway and no variation was therefore observed in the variable. In Alaska and Greenland a score was generated by adding 9 variables measuring standard of housing. This score was further categorised based on its distribution. Worsening standard of housing is illustrated with an increased score. Living in a town or village was not included in the analyses of data from Norway as all participants are by definition rural. However, we included herein living in Finnmark or not, as prior studies [52] have shown that a north–south-based health gradient is relevant to account for in Norway.

### Statistical analyses

The subsistence score and SILA were included in six separate regional- and gender-specific ordinal logistic regressions. Univariate regression and backwards step-wise regression was used to fit the models; covariates included in the final model (Model 2) were significant in at least one stratum. As sample sizes were small, multiple imputation (MI) was executed to improve precision. The complete regression model was applied throughout the imputation process. Wald tests of regression parameters showed no evidence of systematic differences between imputed and non-imputed data (data not shown). Tests of model improvement (e.g. likelihood ratio tests) were not possible as these are not directly applicable to MI results [53]. All statistical analyses were performed using STATA version 12.0 (STATA Corp, College Station, TX).

### Ethics

Detailed information on the project was given to the participants orally and in writing, and written informed consent was obtained before interviews took place. For respondents younger than 18 years, prior written informed consent from parents or legal guardians was obtained. In Norway, the study was accredited by the Norwegian Social Science Data Service and the National Committee for Research Ethics in the Social Sciences and the Humanities. In Alaska, the study was approved by the University of Alaska Institutional Review Board (IRB). In Greenland, approval from the research ethics committee was not obtained, because this is required only for medical research projects. Being responsible for data collection, Statistics Greenland guaranteed an ethical handling of individual data and these rules and regulations ensuring confidentiality for respondents were followed. The survey adheres to the Declaration of Helsinki and to the International Arctic Social Science Association’s (IASSA) Guiding Principles for the Conduct of Research in the Arctic 1998. Representatives of the Inuit Circumpolar Conference, the Sami Council and the Russian Association of Indigenous Peoples of the North have formed advisory boards to oversee the study [54]. Indigenous steering committees approved the final questionnaire [55]. This section is also sited elsewhere [46].

## Results

The distribution of variables by country/region and gender included in this study is presented in Table 1. No significant gender differences in the distributions of SRH, the subsistence score, or SILA were present.

**Table 1:** **Crude distributions of variables by gender and country**/**region**

	**Greenland**		**P**^**a**^	**Alaska**		**P**	**Norway**		**P**
	**Men (%)**	**Women (%)**		**Men (%)**	**Women (%)**		**Men (%)**	**Women (%)**	
Self-rated health			0.340			0.433			0.166
**Very good**/Excellent	**95** (**18**.**7**)	**100** (**18**.**1**)		53 (18.7)	62 (16.4)		45 (21.2)	34 (15.8)	
**Good** / Very good	**303** (**59**.**5**)	**307** (**55**.**5**)		86 (30.4)	108 (28.5)		65 (30.7)	55 (25.6)	
**Fair** / Good	**86** (**16**.**9**)	**115** (**20**.**8**)		81 (28.6)	136 (35.9)		63 (29.7)	69 (32.1)	
**Poor** / Fair	**14** (**2**.**8**)	**23** (**4**.**2**)		50 (17.7)	59 (15.6)		24 (11.3)	39 (18.1)	
**Very poor** / Poor	**3** (**0**.**6**)	**4** (**0**.**7**)		10 (3.5)	14 (3.7)		13 (6.1)	16 (7.4)	
Missing	**8** (**1**.**6**)	**4** (**0**.**7**)		3 (1.1)	-		2 (0.9)	2 (0.9)	
Subsistence score			0.062^b^			0.110^b^			0.063^b^
Mean	0.75	0.70		0.58	0.52		0.86	0.77	
95% CI	0.71-0.79	0.66-0.74		0.52-0.64	0.47-0.57		0.79-0.92	0.70-0.83	
n	498	538		270	373		196	198	
Missing, n (%)	11 (2.2)	15 (2.7)		13 (4.6)	6 (1.6)		16 (7.6)	17 (7.9)	
Language ability (SILA)			0.098			0.936			0.767
Very well	354 (69.6)	388 (70.2)		80 (28.3)	103 (27.2)		131 (61.8)	131 (60.9)	
Relatively well	133 (26.1)	142 (25.7)		27 (9.5)	34 (9.0)		26 (12.3)	30 (14.0)	
With effort	9 (1.8)	16 (2.9)		30 (10.6)	48 (12.7)		23 (10.9)	29 (13.5)	
A few words	10 (2.0)	2 (0.4)		108 (38.2)	150 (39.6)		23 (10.9)	17 (7.9)	
Not at all	2 (0.4)	3 (0.5)		33 (11.7)	42 (11.1)		8 (3.8)	8 (3.7)	
Missing	1 (0.2)	2 (0.4)		5 (1.8)	2 (0.5)		1 (0.5)	-	
Age			0.849			0.327			0.194
16-34 years	165 (32.4)	181 (32.7)		109 (38.5)	140 (36.9)		33 (15.6)	43 (20.0)	
35-59 years	267 (52.5)	296 (53.5)		119 (42.1)	179 (47.2)		117 (55.2)	124 (57.7)	
60-87 years	76 (14.9)	76 (13.7)		54 (19.1)	59 (15.6)		61 (28.8)	47 (21.9)	
Missing	1 (0.2)	-		1 (0.4)	1 (0.3)		1 (0.5)	1 (0.5)	
Education			<0.001			0.341			0.003
Less than high school/vocational school	295 (58.0)	338 (61.1)		85 (30.0)	132 (34.8)		46 (21.7)	33 (15.4)	
High school/vocational school	184 (36.2)	141 (25.5)		183 (64.7)	226 (59.6)		81 (38.2)	59 (27.4)	
University	28 (5.5)	61 (11.0)		12 (4.2)	20 (5.3)		85 (40.1)	120 (55.8)	
Missing	2 (0.4)	13 (2.4)		3 (1.1)	1 (0.3)		-	3 (1.4)	
Smoking			0.017			<0.001			0.668
Does not smoke	143 (28.1)	145 (26.2)		107 (37.8)	142 (37.5)		140 (66.0)	134 (62.3)	
Occasionally	39 (7.7)	36 (6.5)		31 (11.0)	29 (7.7)		13 (6.1)	11 (5.1)	
Half pack or less daily	175 (34.4)	232 (42.0)		66 (23.3)	142 (37.5)		29 (13.7)	38 (17.7)	
More than half pack daily	117 (23.0)	92 (16.6)		67 (23.7)	52 (13.7)		22 (10.4)	20 (9.3)	
Missing	35 (6.9)	48 (8.7)		12 (4.2)	14 (3.7)		8 (3.8)	12 (5.6)	
Chronic problem			0.007			0.814			0.007
Yes	58 (11.4)	96 (17.4)		37 (13.1)	47 (12.4)		49 (23.1)	77 (35.8)	
No	433 (85.1)	440 (79.6)		239 (84.5)	324 (85.5)		154 (72.6)	133 (61.9)	
Missing	18 (3.5)	17 (3.1)		7 (2.5)	8 (2.1)		9 (4.25)	5 (2.3)	
Living in			0.015			0.049			**0**.**284**
Village/**Finnmark**	201 (39.5)	178 (32.2)		146 (51.6)	166 (43.8)		**114** (**53**.**8**)	**127** (**59**.**1**)	
Town/**Not in Finnmark**	308 (60.5)	375 (67.8)		137 (48.4)	213 (56.2)		**98** (**46**.**2**)	**88** (**40**.**9**)	
Missing	-	-		-	-		-	-	
Housing condition			0.114			0.372			-
0-1	157 (30.8)	190 (34.4)		148 (52.3)	197 (52.0)		-	-	
0-2	136 (26.7)	157 (28.4)		63 (22.3)	95 (25.1)		-	-	
4-9	155 (30.5)	137 (24.8)		63 (22.3)	68 (17.9)		-	-	
Missing	61 (12.0)	69 (12.5)		9 (3.2)	19 (5.0)				

^a^ Fisher’s exact chi^2^ test on non-missing data only.

^b^ Two-sided t-test.

The crude distributions of SRH by the included variables are presented in Table 2. A close to significant relationship between poorer SRH and an increased subsistence score was observed among men in Alaska (p=0.08) and Greenland (0.07). In Greenland, a significant relationship and a similar trend was observed between SRH and SILA for both men (p=0.02) and women (p=0.04), while a somewhat reversed relationship was detected in Alaska (p<0.01).

**Table 2:** **Crude distribution of self**-**rated health by country**, **gender and relevant confounders**

**Self**-**rated health (%)**	**Greenland**^**a**^						**Alaska**^**b**^						**Norway**^**b**^					
	**Men**			**Women**			**Men**			**Women**			**Men**			**Women**		
	**0**	**1**	**2**	**0**	**1**	**2**	**0**	**1**	**2**	**0**	**1**	**2**	**0**	**1**	**2**	**0**	**1**	**2**
Subsistence score																		
0-0.54	24.2	53.9	21.8	20.6	51.4	28.0	23.2	28.2	48.6	17.8	29.8	52.4	25.0	26.9	48.1	22.2	20.6	57.1
0.55-1	18.8	64.1	17.2	19.1	58.4	22.5	14.3	41.7	44.1	13.3	25.6	61.1	25.0	32.9	42.1	13.4	23.2	63.4
1.01-3	13.3	62.2	24.4	12.0	61.1	26.9	15.9	22.7	61.4	17.2	31.0	51.7	19.7	33.3	47.0	15.7	35.3	49.0
P-value^c^			0.07			0.19			0.08			0.70			0.86			0.26
Language ability (SILA)																		
Very well – **Very well**/**relatively well**	22.0	58.9	19.1	20.7	54.2	25.1	**25**.**5**	**19**.**8**	**54**.**7**	**14**.**6**	**19**.**0**	**66**.**4**	24.6	30.8	44.6	13.9	21.5	64.6
Not very well – **Not well**	11.9	64.2	23.8	11.8	60.9	27.3	**15**.**2**	**38**.**0**	**46**.**8**	**17**.**5**	**34**.**2**	**48**.**3**	16.5	31.7	51.9	19.3	32.5	48.2
P-value			0.02			0.04			0.00			0.00			0.35			0.06
Age																		
16-34 years	20.4	66.1	13.6	20.8	66.9	12.4	16.7	37.0	46.3	20.0	35.0	45.0	30.3	42.4	27.3	16.7	38.1	45.2
35-59 years	20.8	60.8	18.5	18.3	56.3	25.4	22.2	32.5	45.3	17.9	27.9	54.2	21.6	31.0	47.4	16.9	25.8	57.3
60-87 years	8.2	48.0	43.8	11.8	29.0	59.2	16.7	14.8	68.5	3.4	15.3	81.4	16.7	25.0	58.3	10.9	15.2	73.9
P-value			0.00			0.00			0.02			0.00			0.08			0.08
Education																		
< High school/vocational school	16.6	61.4	22.1	16.8	53.0	30.2	9.5	25.0	65.5	14.4	18.9	66.7	20.0	20.0	60.0	6.1	21.2	72.7
High school/vocational school	22.0	61.0	17.0	19.2	62.4	18.4	22.4	33.9	43.7	17.3	32.3	50.4	15.0	31.3	53.8	13.8	17.2	69.0
University	25.0	46.4	28.6	26.2	57.4	16.4	33.3	25.0	41.7	20.0	50.0	30.0	28.2	36.5	35.3	20.2	31.1	48.7
P-value			0.21			0.02			0.00			0.00			0.03			0.03
Smoking																		
Do not smoke	17.9	59.2	22.9	25.6	54.4	20.0	22.6	31.4	46.0	18.7	31.0	50.3	19.2	34.4	46.4	20.8	26.4	52.8
Half pack or less daily	21.8	58.1	20.1	13.0	58.3	28.7	18.2	30.3	51.5	15.5	31.0	53.5	25.5	21.6	52.9	7.0	19.3	73.7
More than half pack daily	17.4	65.2	17.4	12.0	55.4	32.6	10.5	28.4	61.2	13.5	17.3	69.2	-	-	-	-	-	-
P-value			0.63			0.00			0.21			0.18			0.21			0.01
Chronic problem																		
Yes	15.8	28.1	56.1	4.2	35.4	60.4	13.5	5.4	81.1	4.3	8.5	87.2	2.0	18.4	79.6	2.7	12.0	85.3
No	19.7	65.0	15.3	22.0	59.9	18.1	20.1	34.7	45.2	18.5	31.5	50.0	28.6	35.1	36.4	24.1	33.8	42.1
P-value			0.00			0.00			0.00			0.00			0.00			0.00
Living in																		
Village/**Finnmark**	18.6	60.3	21.1	17.1	58.3	24.6	18.1	27.8	54.2	15.7	24.7	59.6	**28**.**1**	**31**.**6**	**40**.**4**	**15**.**0**	**22**.**1**	**63**.**0**
Town/ **Not in Finnmark**	19.2	60.6	20.2	18.7	54.8	26.5	19.9	33.8	46.3	16.9	31.5	51.6	**13**.**5**	**30**.**2**	**56**.**3**	**17**.**4**	**31**.**4**	**51**.**2**
P-value			0.98			0.78			0.41			0.26			0.02			0.20
Housing condition																		
0-1	23.1	61.5	15.4	22.1	52.6	25.3	20.3	37.2	42.6	18.8	32.5	48.7	-	-	-	-	-	-
0-2	14.8	63.7	21.5	18.5	59.9	21.7	19.4	27.4	53.2	15.8	22.1	62.1	-	-	-	-	-	-
4-9	18.4	57.2	24.3	14.7	52.2	33.1	17.7	21.0	61.3	11.8	26.5	61.8	-	-	-	-	-	-
P-value			0.17			0.14			0.11			0.15			-			-

^a^ Self-rated health coded: 0) Very good, 1) Good, 2) Fair/Poor/Very Poor.

^b^ Self-rated health coded: 0) Excellent , 1) Very good, 2) Good/Fair/Poor.

^c^ Fisher’s exact chi^2^ test on non-imputed data.

Multivariable analyses are displayed in Table 3. Acculturation significantly predicted poorer SRH in Greenland, and the relative effects of acculturation were stronger for men than for women, though this modification of effects was not significant (data not shown). An increased subsistence score gave an OR of 2.32 (P<0.001) for reporting poorer SRH among Greenlandic men, while an increased score for Greenlandic women generated an OR of 1.71 (P=0.01). Poorer SILA produced an OR of 1.59 in men (p=0.03) and 1.43 in women (p=0.07). In Alaska, no evidence of acculturation effects was detected among Iñupiaq men. Among Iñupiaq women, an increased score represented an increased odds of 73% (p=0.026) for reporting poorer SRH. No significant effects of acculturation were detected in Norway. However, SILA was close to significant (p=0.068) among Sami men, thus suggesting a substantial effect of acculturation on SRH (OR=1.74).

**Table 3:** **Adjusted odds ratios**^**a**^**for reporting poorer self**-**rated health**^**b**^

**Countries and variables**	**Model 1**^**c**^			**Model 2**^**d**^		
	**OR**	**P-value**	**95% CI**	**OR**	**P-value**	**95% CI**
Greenland – men (n=509)						
Subsistence score	2.31	<0.001	1.49-3.56	2.32	<0.001	1.50-3.58
Language ability (SILA)						
Very well	1	Ref.	Ref.	1.	Ref.	Ref.
Not very well	1.58	0.033	1.04-2.42	1.59	0.032	1.04-2.43
Greenland – women (n=553)						
Subsistence score	1.67	0.014	1.11-2.51	1.71	0.010	1.13-2.56
Language ability (SILA)						
Very well	1	Ref.	Ref.	1.	Ref.	Ref.
Not very well	1.42	0.075	0.97-2.09	1.43	0.072	0.97-2.10
Alaska – men (n=284)						
Subsistence score	1.38	0.226	0.82-2.34	1.39	0.215	0.83-2.35
Language ability (SILA)						
Very well/relatively well	1	Ref.	Ref.	1	Ref.	Ref.
Not well	1.39	0.264	0.78-2.48	1.39	0.263	0.78-2.49
Alaska – women (n=380)						
Subsistence score	1.73	0.028	1.06-2.81	1.73	0.026	1.07-2.82
Language ability (SILA)						
Very well/relatively well	1	Ref.	Ref.	1	Ref.	Ref.
Not well	0.81	0.428	0.48-1.37	0.81	0.427	0.48-1.37
Norway – men (n=212)						
Subsistence score	0.82	0.564	0.42-1.60	0.81	0.524	0.42-1.56
Language ability (SILA)						
Very well	1	Ref.	Ref.	1	Ref.	Ref.
Not very well	1.31	0.428	0.67-2.57	1.74	0.068	0.96-3.15
Norway – women (n=215)						
Subsistence score	1.03	0.935	0.56-1.90	1.01	0.972	0.55-1.86
Language ability (SILA)						
Very well	1	Ref.	Ref.	1	Ref.	Ref.
Not very well	0.84	0.634	0.40-1.74	0.72	0.302	0.38-1.35

^a^ Multivariable ordinal logistic regression on imputed data.

^b^ SRH coded in Greenland: 0) Very good, 1) Good, 2) Fair/Poor/Very Poor.

SRH coded in Alaska and Norway: 0) Excellent , 1) Very good, 2) Good/Fair/Poor.

^c^ Greenland and Alaska: controlling for age, education, smoking, chronic problem, housing condition, living in village or town.

Norway: controlling for age, education, smoking, chronic problem, living in Finnmark or not.

^d^ Greenland and Alaska: controlling for age, education, smoking, chronic problem, housing condition.

Norway: controlling for age, education, smoking, chronic problem.

Overall, no modifying effect of the subsistence score by levels of SILA was detected (data not shown). Furthermore, we found no evidence of education confounding or modifying the effect of acculturation (data not shown).

## Discussion

In this article, we have presented the effects of acculturation on SRH among the Kalaallit of Greenland, the Iñupiat of Alaska, and Sami of Norway. This study is the first comparative analysis of acculturative effects among these indigenous peoples, and the first study of this kind ever in the adult Sami population in Norway. Acculturation was positively and significantly associated with poorer SRH in Greenland, and in Alaska among Iñupiaq women.

The observed relationship between acculturation and SRH may perhaps be explained by the aforementioned effects of colonialism and modernisation. A more sedentary lifestyle with increasing consumption of store-bought foods is a plausible explanation. Also, acculturative stress and related poor health behaviours such as alcohol and substance abuse are contributing factors. Extensive research has demonstrated that acculturation can have both negative and protective effects depending on the outcome of interest. Therefore, a relationship between acculturation and SRH gives a good indication of the overall effects of acculturation on composite health among the Sami, Iñupiat and Kalaallit.

That we could not observe a significant relationship between acculturation and SRH among the Sami may be because of the relatively early governmental focus on nation-wide equality in health care access in Norway. Financially, the Norwegian state contributed more to the reconstruction of health care infrastructure in Finnmark than elsewhere in the immediate post-war years, from which the rather large Sami population in the county have benefited from. The subsequent large scale regionalization of higher education in Norway in the 1970s also helped along the education of Sami-speaking physicians and other professionals needed in health care and other industries in Sami settlement areas [56]. This development may also explain the relatively high SES among the Sami today. However, the observed effect of SILA in Sami men was close to significant and may not be disregarded as random sampling variation. Language ability has shown to be a good indicator of acculturation in previous studies (see for example Bjerregaard et al. [36]) and among Kalaallit men in this study. Lack of indigenous language can indicate a reduced possibility of inclusion in the indigenous culture, or perhaps an orientation away from it. This includes adaptation to majority customs such as alcohol use [2].

Prior studies argue that Inuit men have to a greater extent than women experienced more problems integrating into the modern society; Inuit women have for example adapted more comfortably to higher education and wage employment [24,57]. This development is also observed among Sami women [58]. Though we found higher education levels among women in this study, we did not find any evidence suggesting stronger effects of acculturation in men, as did not Bjerregaard et al. [36] in their study on acculturation and mental health.

That we could not find a relationship between acculturation and SRH among Iñupiaq men may be because our operationalization of acculturation measured the phenomenon poorly in this stratum; acculturation is a multidimensional concept and our linear approach may thus have been unable to measure true acculturation effects among these men (see below). Also, considering the p-values of about 20%, we may because of relatively small numbers in the strata of Iñupiaq men, have been unable to pick up on potential true effects.

Important to note is that the involved populations differ greatly in terms of the legal framework conveying indigenous autonomy and self-determination [59-62], thus generating discrepancies between the countries with regard to the premise for developing and securing culture, languages, and land and subsistence rights. Also, the Sami are in minority in most municipalities in Sápmi (apart from the municipalities of Karasjok, Kautokeino and Nesseby), while the Iñupiat and Kalaallit are in majority in their regions. The degree and pace of acculturation is of course affected by such demographic incentives. Differences in autonomy and self-determination may of course also affect the impact of acculturation and, subsequently the observed effects of acculturation on health in these populations. However, the self-governing arrangements in the Arctic are of comparatively recent date; further comparative studies must be executed to better understand the significance of the various political arrangements for human development [63], and health.

### Limitations

An essential limitation in the study was that the response options in our dependent variable, SRH, differed in wording in Greenland (*Very good* through *Very poor*) compared with Norway and Alaska (*Excellent* through *Poor*). As the distance between the categories differed due to variation in wording [64], the frame of reference was unstandardized; the distributions of SRH are thus incomparable [65]. What still was possible, though, was to analyse the direction of the associations. Significant relationships and careful assessments of the overall strength of the associations gave a general impression of the overall effects of acculturation on SRH among the involved populations. Also, SILA was coded differently (see methods). The same limitations with regard to the assessments of effect sizes were relevant herein.

While SRH appears to be a valid measure for assessing health, there are potential cultural differences in how SRH is conceptualised and the determinants that factor into self-assessments of health [66]. Caution must in that regard be shown in terms of comparing the relative strength of the association among these populations. Overall consistency of meaning in the questionnaire was however secured during the design-stage of SLiCA [55].

#### Acculturation - a multidimensional concept

The linear acculturation scales have come under criticism as they represent a simplification of the acculturation process and rely on an assumption of two distinct cultures [47]. The multidimensional models such as Berry’s [50] are designed to identify adaptations beyond the dichotomy of high versus low acculturation. These more complex scales, however, still also rely on the notion of culturally distant groups. While individuals may be conceptualised within a multidimensional framework of adaptations, the assumption of two culturally distant groups nevertheless still persist [47]. Furthermore, the criticism of the linear scales has also been related to the fact that many scales have been produced and applied without prior metric analysis of scale reliability or factor structure [48]. As aforementioned, our factor analyses produced consistent results displaying very strong internal consistency in the items, thus suggesting that our approach is an appropriate substitute. As we were unable to measure multiple dimensions, our estimates become thus aggregate or overall effect measures of acculturation. The comparability of our study with acculturation studies using Berry’s model is thus perhaps not ideal.

#### Confounding

The overall comparability in this study is somewhat reduced as the populations differ with regard to living condition and the distribution of general risk factors; comparing the associations between acculturation and SRH may be flawed if the Inuit and Sami differ with regard to the distribution of confounding factors. This may however be statistically corrected for by adjusting for known and relevant confounding variables [67]. We have assessed and accounted for several potential major confounders, but not all.

The notion that acculturation is synonymous with ill health may arise from failure to control for the negative effects of discrimination and SES, which often co-exist with acculturation [32,47,48,68]. Low SES may in some instances even work as a dimension of acculturation and in other function as an effect modifier [68]. We found no evidence of education confounding or modifying the effect of acculturation. Nevertheless, using education as a proxy for SES in this study is indeed challenging as access to education and the educational systems differ among the Inuit and the Sami, and it is likely that education is a more appropriate proxy in Norway than in Alaska and Greenland. Studies show that conventional SES such as education attainment cannot be directly applied to Inuit populations [36]. Residual SES confounding may therefore be an issue in our study, which may damage the internal validity. However, it is important to note that education levels among the Inuit are improving rapidly [9], thus strengthening the validity of education as a proxy for SES. Nevertheless, one still knows little about how SES plays into the relationship of acculturation and health. More research is needed to assess possible confounding, mediating or modifying effects.

As information on discrimination was not included in the questionnaire, we have been unable to control for its assumed effect. Our estimates may in this regard be biased away from the null [68].

The association between acculturation and health status is conceptually thought to be mediated by health care use [69]. The Inuit of Alaska and Greenland, and the Sami are provided publicly funded health care [70,71]. What first and foremost distinguish Alaska and Greenland from Norway in terms of health care, are the enormous distances, poorly developed infrastructure, and the climatic conditions. A study in Canada among Inuit showed that education and remoteness are important factors in terms of access to health care [72]. We have thus controlled for these variables; education and remoteness however do not exclusively explain variation in health care use. Residual confounding in this regard may also limit our analysis. Furthermore, language-based and culture sensitive health care service is offered to a varying degree in these countries which of course affect both access to services but also the degree to which people are inclined to use them.

Overall, because of bias due to discrepancies in wording, categorisation and cultural conceptualisation of SRH, and potential confounding, we cannot conclude convincingly on the relative strength of the effect estimates. It is plausible that residual confounding is influential in these analyses; we are however convinced that enough confounding is accounted for in terms of controlling any distortion of qualitative character potentially affecting the direction of the associations. Also, comparison of effect sizes in this study is not recommended as study efficiency and precision is affected by small sample sizes.

## Conclusions

This study shows that aggregate acculturation is a strong risk factor for poorer SRH among the Kalaallit of Greenland and female Iñupiat of Alaska, but our cross-sectional study design does not allow any conclusion with regard to causality. Limitations with regard to wording, categorisations, assumed cultural differences in the conceptualisation of SRH, and confounding effects of health care use, SES and discrimination, make it difficult to appropriately assess how strong this effect is though. Acculturation is indeed a process that takes place over time [3]; longitudinal research and large samples are therefore required to examine the effect of acculturation on SRH within multiple dimensions among these populations, while simultaneously exploring how SES may play into this relationship.

## Abbreviations

IASSA: International Arctic Social Science Association; IRB: University of Alaska Institutional Review Board; MI: Multiple Imputations; OR: Odds Ratio; SES: Socioeconomic Status; SILA: Spoken Indigenous Language Ability; SLiCA: Survey of Living Conditions in the Arctic: Inuit, Sami and the indigenous peoples of Chukotka; SRH: Self-Rated Health; STATA: STATA: Data Analysis and Statistical Software; 95% CI: 95% Confidence Interval.

## Competing interests

The authors declare that they have no competing interests.

## Authors’ contributions

BME carried out the statistical analysis and drafted the manuscript. TB executed the multiple imputation of missing data and participated in the statistical analysis. MM, KLH and ARB helped to draft the manuscript, while also MM assisted the statistical analysis. All authors read and approved the final manuscript.

## Pre-publication history

The pre-publication history for this paper can be accessed here:

http://www.biomedcentral.com/1471-2458/12/948/prepub
